# T2-FLAIR hyperintensities in the inferior cerebellar peduncles and their association with clinical symptoms, molecular and MRI markers in male *FMR1* premutation carriers

**DOI:** 10.3389/fnmol.2026.1720370

**Published:** 2026-02-16

**Authors:** Andrea Elias-Mas, Irene Paracuellos-Ayala, Jun Yi Wang, Kyoungmi Kim, Flora Tassone, Andrea Schneider, David Hessl, Susan M. Rivera, Randi J. Hagerman

**Affiliations:** 1 Department of Radiology, Hospital Universitari Mútua de Terrassa, (HUMT), Terrassa, Spain; 2 Institute for Research and Innovation Parc Taulí (I3PT), Sabadell, Spain; 3 Genetics Doctorate Program, Universitat de Barcelona (UB), Barcelona, Spain; 4 Center for Mind and Brain, University of California, Davis, Davis, CA, United States; 5 Department of Public Health Sciences, School of Medicine, University of California, Davis, Sacramento, CA, United States; 6 MIND Institute, University of California, Davis, Sacramento, CA, United States; 7 Department of Biochemistry and Molecular Medicine, School of Medicine, University of California, Davis, Sacramento, CA, United States; 8 Department of Psychiatry and Behavioral Sciences, School of Medicine, University of California, Davis, Sacramento, CA, United States; 9 Department of Psychology, University of Maryland, College Park, MD, United States; 10 Department of Pediatrics, University of California Davis Medical Center, Sacramento, CA, United States

**Keywords:** ataxia, FMR1 premutation, fragile X-associated tremor/ataxia syndrome, FXTAS, inferior cerebellar peduncle, MRI, white matter

## Abstract

**Background and objectives:**

*FMR1* premutation carriers (55–200 CGG repeats) are at risk of developing fragile X-associated tremor/ataxia syndrome (FXTAS), a neurodegenerative disorder associated with motor and cognitive impairment. Bilateral hyperintensities of the middle cerebellar peduncles (MCP sign) are the major radiological hallmarks of FXTAS. The inferior cerebellar peduncles (ICP) contain fibers related to proprioception and vestibular functions (such as the rostral and posterior spinocerebellar tracts and the juxta restiform body), which are clinically associated with cerebellar gait ataxia, a major clinical criterion for FXTAS diagnosis. However, the ICP hyperintensity has yet to be studied in FXTAS.

**Methods:**

We evaluated 588 MRI scans (mean 2.05 visits/participant) from 202 male premutation carriers (164 with FXTAS and 38 without FXTAS at last visits) and 85 controls. Two radiologists, independently, rated as absent or present the signal of the right and left ICP in T2-Fluid-attenuated inversion recovery (FLAIR) scans. Mixed-effects models were used for statistical analysis adjusting for age.

**Results:**

Only carriers with FXTAS revealed ICP hyperintensities at last visits. Furthermore, ICP hyperintensity was associated with brain atrophy, increased white matter disease, the MCP sign, FXTAS stage, abnormal gait, lower cognitive functioning and faster age-related increase in anxiety and depression scores. Finally, carriers with ICP hyperintensities had significantly higher CGG repeat length than carriers without ICP hyperintensities.

**Discussion:**

This study describes ICP hyperintensity as a new potential radiological finding in FXTAS, suggests involvement of the vestibulo-cerebellar, rostral, and posterior spinocerebellar tracts, and the vestibular system in FXTAS physiopathology, and reinforces the association of CGG expansion in the range of brain changes seen in FXTAS.

## 1 Introduction

Fragile X-associated tremor/ataxia syndrome (FXTAS) is a late-onset neurodegenerative disorder affecting carriers of the Fragile X Messenger Ribonucleoprotein 1 (*FMR1*) gene premutation alleles (55–200 CGG repeats) who exhibit excessive *FMR1* mRNA levels but normal or slightly reduced *FMR1* protein (FMRP) level (Kenneson et al., 2001). Though the prevalence of *FMR1* premutation is 1:400–850 in men and 1:150–300 in women, only 40%–75% of males and 16%–20% of females with the premutation will develop FXTAS. Symptoms typically start in the 60 s, with intention tremor and cerebellar ataxia - both considered major clinical diagnostic criteria, and potentially cognitive decline. Parkinsonism, peripheral neuropathy, lower limb proximal muscle weakness, autonomic and vestibular dysfunction, and psychiatric manifestations, such as anxiety and depression, have also been described (Hagerman and Hagerman, 2016).

Radiological signs of FXTAS include white matter hyperintensities (WMHs) on T2-weighted MRI sequences, particularly in the middle cerebellar peduncle (“the MCP sign,” a major radiological diagnostic criterion), brainstem, corpus callosum, and cerebral deep white matter. Enlarged ventricles and generalized brain atrophy are frequently observed in FXTAS (Brunberg et al., 2002; Wang et al., 2017). Both motor and cognitive symptoms, together with typical radiological findings, lead to a hypothesis that individuals with FXTAS have an impairment of the cerebello-basal ganglia-thalamo-cortical network (Shelton et al., 2017).

Some of the most prevalently affected brain structures in FXTAS are the MCPs, which show hyperintensities on T2-weighted MRI sequences, detected in 58%–82% of males and 11%–13% of females with FXTAS (Brunberg et al., 2002; Adams et al., 2007; Renaud et al., 2015; Schneider et al., 2020). The MCPs are the largest among the three cerebellar peduncles. They originate in the lateral pons and extend laterally to the superior and inferior cerebellar peduncles (SCPs and ICPs), sharing a close anatomical relationship, especially with the ICPs, whose anatomical boundaries are less well-defined on MRI scans acquired with current imaging techniques (Jones et al., 2013; Paracuellos-Ayala et al., 2025). The MCPs are considered the main afferent pathways to the cerebellum. Their notable components are the pontocerebellar projections, which transmit information from the contralateral cerebral cortex, via the pontine nuclei, to the cerebellum, contributing to motor planning, cognition, and language functions (Jones et al., 2013).

Microscopic examination of the MCPs of individuals with FXTAS revealed spongiosis reflecting degeneration (Greco et al., 2006), and diffusion tensor imaging (DTI) showed reductions in both connectivity and fractional anisotropy (FA) (Hashimoto et al., 2011; Wang et al., 2013; Filley et al., 2015). Moreover, spectroscopic abnormalities and reduced FA on the MCPs of individuals with FXTAS correlated with executive dysfunction (Filley et al., 2015) and FXTAS severity (Hashimoto et al., 2011).

Interestingly, proprioceptive information from the trunk, upper and lower limbs, enters the cerebellum through the SCPs (rostral and anterior spinocerebellar tracts) and ICPs (rostral and posterior spinocerebellar tracts), but not through the MCP. Similarly, information from the reticular and visual systems reaches the cerebellum via the ICPs, through the reticulocerebellar and olivocerebellar tracts (Jones et al., 2013; Paracuellos-Ayala et al., 2025). These systems, along with proprioceptive input, contribute to maintaining postural balance and coordinating movements such as walking. Their anatomical pathways within the ICPs, explain why inflammatory, infectious, or ischemic insults to the ICP can result in postural imbalance and impaired walking ability (Choi et al., 2015; Kim et al., 2019).

Although the ICPs may play a key role in the ataxia observed in individuals with FXTAS, few studies have specifically investigated their involvement in FXTAS symptomatology. To date, only one study obtained inconsistent results between two different types of DTI analyses of the ICP in male *FMR1* premutation carriers, suggesting its involvement in FXTAS while highlighting the need for further studies using more refined imaging techniques (Hashimoto et al., 2011). On the contrary, another study showed associations between decreased ICP fiber integrity and higher methylation levels in the *FMR1* gene in female premutation carriers (Shelton et al., 2017). Therefore, the scarce published evidence appears to indicate that the ICPs may be affected in FXTAS. However, unlike the MCPs, which are well-documented to be affected in FXTAS and typically appear hyperintense on T2-weighted sequences, no studies have examined whether the ICPs also exhibit T2 hyperintensities in FXTAS.

Having in mind that cerebellar gait ataxia is considered a major clinical diagnostic criterion in FXTAS and, given the anatomical and functional roles of both the MCPs and ICPs in motor control, in this study we hypothesized that male premutation carriers with FXTAS exhibit a higher incidence of ICP T2 hyperintensities compared to carriers without FXTAS and non-carrier controls. We further hypothesized that these ICP signal abnormalities are associated with motor and cognitive dysfunction, MRI signs of neurodegeneration, and CGG repeat length, potentially offering new insights into FXTAS pathophysiology.

## 2 Materials and methods

### 2.1 Research participants and outcome measurements

The study was conducted following procedures approved by the University of California Davis Institutional Review Board (protocol code 215292 and date of approval 17 July 2019). Written informed consent was obtained from all participants involved in the study or from their caregivers.

T2-weighted fluid-attenuated inversion recovery (FLAIR) MRI scans were acquired from male participants (2007–2023). Because the youngest with MCP/ICP hyperintensities was 48, only participants aged ≥ 40 were included.

A total of 588 MRI scans were available from 287 males aged 40–85 years. These include 202 premutation carriers, of whom 84 had 1 visit, 60 had 2 visits, 24 had 3 visits, 21 had 4 visits, 8 had 5 visits, 2 had 6 visits, 1 had 8 visits, 1 had 9 visits and 1 had 10 visits (mean = 2.17 visits, SD = 1.48 visits). The controls were 85 males with normal *FMR1* alleles, of whom 59 had 1 visit, 5 had 2 visits, 5 had 3 visits, 15 had 4 visits and 1 had 5 visits (mean = 1.75 visits, SD = 1.23 visits). FXTAS diagnoses made by trained physicians were based on core FXTAS symptoms, which included intention tremor, cerebellar ataxia, cognitive impairment, and WMHs in specific brain regions (Hall et al., 2014; Schneider et al., 2020; Tassone et al., 2023). Carriers with FXTAS diagnosis of “No” were classified as FXTAS negative (PFX-), whereas those with “Possible,” “Probable,” and “Definite” diagnoses were classified as FXTAS positive (PFX+). FXTAS stages were assessed according to physical disability as (1) subtle or questionable tremor and/or balance problems; (2) minor tremor and/or balance problems, with minimal interference in activities of daily living (ADLs); (3) moderate tremor and/or balance problems with significant interference in ADLs; (4) severe tremor and/or balance problems, requiring a cane or walker; (5) daily wheelchair use; and (6) bedridden (Bacalman et al., 2006). Motor impairment was assessed using tandem walk rated as “normal,” “abnormal,” and “unable.” Cognitive functioning was evaluated using Wechsler Adult Intelligence Scale, Third (WAIS III, 166 participants, 254 visits) and Fourth Editions (WAIS IV, 80 participants, 111 visits) and Wechsler Abbreviated Scale of Intelligence, Second Edition (71 participants, 97 visits) (Wechsler, 1997, 2008, 2011). Full scale IQ (FSIQ), Working Memory Index (WMI) and Processing Speed Index (PSI) available from WAIS III and IV were included in the analyses. The Behavioral Dyscontrol Scale-2 (BDS-2), a nine-item measure, was used to assess behavioral and attentional self-regulation in motor control and executive function (Grigsby et al., 1992). Psychological symptoms were evaluated using the Symptom Checklist-90-Revised (Derogatis, n.d.). The T-scores of Global Severity Index (GSI), Anxiety and Depression were included in the analyses.

### 2.2 Molecular genetic data/genotyping

Because both mosaicism and partial methylation of the *FMR1* CGG repeat have been reported in male premutation carriers (Pretto et al., 2014), CGG repeat size and methylation status were examined from genomic DNA isolated from peripheral blood leukocytes, using PCR and Southern blot analysis as previously described (Tassone et al., 1999, 2008; Filipovic-Sadic et al., 2010). The percentage of methylation was measured by densitometry analysis (Tassone et al., 1999). Male carriers with either one or two premutation alleles, and no evidence of methylation, were included. For mosaic carriers (those with two *FMR1* premutation alleles), CGG repeat size was averaged. RNA was purified from 2.5 mL peripheral blood, and *FMR1* mRNA expression levels were measured using quantitative reverse transcription-PCR, as previously (Tassone et al., 2000).

### 2.3 MRI acquisitions and analyses

All participants were scanned on a Siemens Trio 3T MRI scanner (Siemens Medical Solutions, Erlangen, Germany) with either an 8-channel (94 scans) or 32-channel head coil (494 scans). One-millimeter isotropic T1-weighted scans covering the whole brain were collected using the magnetization-prepared rapid gradient-echo (MPRAGE) sequence. We calculated the brain and ventricular volumes following our published methodology (Wang et al., 2017). T2-FLAIR scans were collected in 104 sagittal slices covering the whole brain, with image resolution of 0.95 × 0.95 × 1.9 mm^3^ and 0-mm interslice on reconstructed image. Whole brain WMH volume was quantified using previously reported methods (Wang et al., 2021).

**FIGURE 1:**
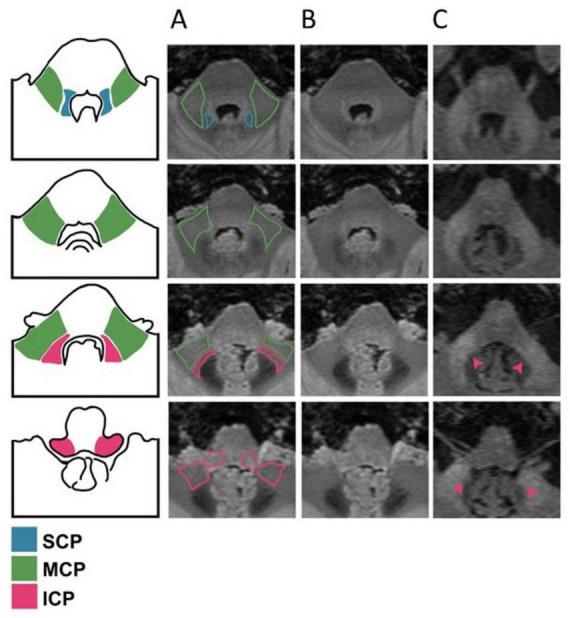
Delineation of the cerebellar peduncles and assessment of signal abnormalities in fragile X-associated tremor/ataxia syndrome (FXTAS). **(A)** Visual delineation of each cerebellar peduncle [superior cerebellar peduncles (SCP) in blue, middle cerebellar peduncles (MCP) in green, inferior cerebellar peduncles (ICP) in pink]. **(B)** Normal signal intensities in a healthy individual without the Fragile X Messenger Ribonucleoprotein 1 (*FMR1*) premutation. **(C)** Hyperintense signals in both the ICP and MCP of an individual with the *FMR1* premutation and FXTAS (PFX+). The pink-headed arrows point to the ICP’s hyperintensities.

Middle cerebellar peduncles and ICP hyperintensities (Figure 1) were assessed by two radiologists. Images were available to the readers, who were blinded to all participants’ data. Hyperintensities were rated as 0 = none, or 1 = present. Cohen’s Kappa for two raters based on the whole dataset were 0.91/0.90 (*p* = 0) for left/right MCP and 0.76/0.77 (*p* = 0) for left/right ICP. The numbers of cases that were initially discordant between the two raters were 24 (4.1%)/25 (4.3%) for left/right MCP and 46 (7.8%)/43 (7.3%) for left/right ICP. In case of disagreement, the images were discussed between the 2 readers to reach consensus.

### 2.4 Statistics

Statistical analyses were performed using the open-source R software, version 4.4.1 (R Foundation for Statistical Computing, Vienna, Austria) (R Core Team, 2024). For each type of outcome families (i.e., MRI, motor/cognition, neuropsychological test scores, and *FMR1* molecular data), the Benjamini-Hochberg’s false discovery rate (BH-FDR) procedure was employed to control for multiple testing (Benjamini and Hochberg, 1995) within each outcome domain. For cross-sectional group comparisons of participant characteristics at last visit of follow-up, linear regression was used for comparing age differences between the groups of controls, PFX- and PFX+. For studying cross-sectional group differences in FSIQ, WMI and PSI at last visit, linear regression was used with age and years of education as covariates. Linear regression was used for cross-sectional comparisons of the scores of GSI, anxiety and depression at last visit between the three groups with age as a covariate. For the ordinal measure BDS-2 at last visits, age was adjusted using linear regression and group comparisons were performed using non-parametric Kruskal-Wallis test, followed by *post hoc* pairwise comparisons using Wilcoxon tests. For other continuous measures with non-normal data including years of education, CGG repeat length and *FMR1* mRNA levels at last visit, Kruskal-Wallis test was conducted to assess group differences, followed by *post hoc* pairwise comparisons using Wilcoxon rank-sum tests. Finally, group differences in proportions of cognitive impairment and emotional distress at last visit were assessed using Fisher’s exact tests.

The occurrences of left and right ICP hyperintensities at last visit were compared between the groups using Fisher’s exact tests. The association between ICP and MCP hyperintensities at last visit were assessed using Pearson’s chi-square test. Individual continuous outcome measures (i.e., whole brain volume, whole brain WMH volume, ventricular volume, FXTAS stage, tandem walk, FSIQ, WMI, PSI, BDS-2, GSI, anxiety, depression, and *FMR1* mRNA level) were modeled by left or right ICP hyperintensity rating using linear mixed-effects models, separately in PFX- and PFX+. These mixed-effect models included fixed effects for age at brain scan, left or right ICP hyperintensity rating, and interaction between age at brain scan and ICP hyperintensity rating, and a random intercept for participant. Analysis of residuals was performed to validate the underlying assumptions of the regression models prior to statistical inference. Volumes of WMHs and ventricles and *FMR1* mRNA level were log transformed prior to statistical analyses to meet the normality assumption. Missing data were excluded from the analyses. Years of education was used as a covariate in mixed-effect models involving FSIQ, WMI and PSI. Brain scaling factor, scanner software version, and head coil type were added as covariates in mixed-effect models involving volumes of the whole brain, WMHs, and ventricles to account for individual differences in cranial size (Buckner et al., 2004). Group differences in CGG repeat length between PFX + with and without ICP hyperintensities at last visit were compared using linear regression model, including age, scanner software, head coil as covariates. Finally, we used time-varying effect models to explore changes in the relationships of ICP and MCP hyperintensities with FXTAS stage over the age range (Tan et al., 2012).

## 3 Results

### 3.1 Participant characteristics

Table 1 shows descriptive statistics of basic and demographic characteristics of our participants at their last visits. The PFX+ (29/34/101 with “Possible”/“Probable”/“Definite” FXTAS diagnosis) were older than non-carrier controls and PFX- (PFX+ vs. controls/PFX+ vs. PFX−: β = 4.67 ± 1.14/7.71 ± 1.54, *p* < 0.001). Years of education were also different among the three groups (chi-squared = 16.2, *p* < 0.001; pairwise comparisons PFX− > controls: Wilcoxon *p* = 0.024; PFX+ < controls: Wilcoxon *p* < 0.001). As expected, PFX+ performed worse in tandem walk than both controls and PFX− after adjusted for age (PFX+ vs. controls/PFX+ vs. PFX−: β = 0.97 ± 0.10/0.99 ± 0.13, *p* < 0.001).

**TABLE 1:** Characteristics of participants at last visits.

Groups	Controls	No FXTAS	FXTAS
Age: mean (SD) [range] [*N*]	63.6 (9.4) [40–81] [85]	60.5 (11.0) [40–82] [38]	68.2 (7.4) [47–85] [164]*^
Education (y): median (IQR) [range] [*N*]	16 (3) [6–26] [66]	18 (4) [6–24] [36]*	16 (4) [8–25] [143]^
Tandem walk: abnormal%, unable% [*N*]	13.5%, 2.7% [74]	10.8%, 0% [37]	26.9%, 49.4% [156]*^
Full scale IQ: mean (SD) [range] [*N*]	121.6 (15.0) [90–153] [68]	123.3 (14.6) [91–148] [35]	106.2 (17.2) [62–147] [147]*^
FSIQ ≤ 80:%	0%	0%	9.5%*
WMI: mean (SD) [range] [*N*]	113.2 (15.9) [82–144] [61]	117.4 (17.3) [90–147] [28]	103.7 (16.8) [66-150] [145]*^
WMI ≤ 80:%	0%	0%	9.7%*
PSI: mean (SD) [range] [*N*]	112.1 (16.8) [79–150] [62]	112.9 (13.8) [86–140] [28]	93.4 (17.1) [56–143] [118]*^
PSI ≤ 80:%	2.0%	0%	20.3%*^
BDS-2: median (IQR) [range] [*N*]	23 (5) [17–27] [71]	23 (4.5) [13–27] [35]	19 (8) [2–27] [157]*^
BDS-2 < 14:%	0%	2.9%	24.2%*^
GSI: mean (SD) [range] [N]	53.3 (11.9) [30–79] [65]	52.5 (9.7) [30–68] [34]	56.3 (10.6) [30–81] [136]*^
GSI ≥ 63:%	24.6%	14.7%	29.4%
Anxiety: mean (SD) [range] [*N*]	50.4 (11.0) [40–81] [65]	50.8 (9.3) [40-66] [34]	53.3 (10.6) [40–79] [136]*^
Anxiety ≥ 63:%	13.8%	8.8%	21.3%
Depression: mean (SD) [range] [*N*]	54.2 (11.9) [38–81] [65]	53.5 (10.7) [38–81] [34]	57.4 (11.3) [38–81] [136]*^
Depression ≥ 63:%	23.1%	14.7%	27.9%
CGG repeat: median (IQR) [range] [*N*]	30 (3) [19–43] [83]	73.5 (25.8) [55–183] [38]*	90 (22) [57–141] [163]*^
*FMR1* mRNA: median (IQR) [range] [*N*]	1.31 (0.42) [0.9–1.9] [60]	2.09 (0.42) [1.5–6.6] [22]*	2.63 (0.68) [1.6–5.6] [117]*^

*Significantly different from controls at *p* ≤ 0.05. ^Significantly different from PFX- at *p* ≤ 0.05.

For cognitive impairment, FSIQ, WMI and PSI were lower in the PFX+ group compared with the control group and the PFX− group after adjusting for age and years of education (β = −18.5 ± 2.71 to −8.15 ± 2.73, *p* < 0.001–0.003). BDS-2 was lower in the PFX+ group compared with both control and PFX− groups after adjusting for age (chi-squared = 35.4, *p* < 0.001; pairwise comparisons with control/PFX−: Wilcoxon *p* < 0.001). BDS-2 scores were < 14, indicating impairment, in one PFX− (2.9%) and 39 PFX+ (24.2%) at their last visits but not in controls. Fifteen PFX+ (9.5%) showed major impairment in FSIQ (≤80) at their last visits. Seventeen PFX+(10.7%) showed impaired working memory (WMI ≤ 80) and 33 PFX+ (20.3%) exhibited impaired processing speed (PSI ≤ 80) at their last visits. Only one control showed PSI ≤ 80, while no PFX- showed such scores.

For psychological symptoms, GSI, anxiety and depression scores were higher in the PFX+ group compared with controls and the PFX- group (β = 4.05 ± 1.77 to 5.22 ± 2.13, *p* = 0.009–0.044).

As expected, both CGG repeat length and *FMR1* mRNA expression level were higher in the premutation carrier group compared with the control group (CGG/mRNA: chi-squared = 186.3/127.6, *p* < 0.001; Wilcoxon *p* < 0.001). PFX+ also showed higher CGG repeat length and mRNA level than PFX- (CGG/mRNA: Wilcoxon *p* < 0.001/0.002).

### 3.2 ICP hyperintensities

At last visit, the occurrences of left and right ICP hyperintensities were higher in PFX+, 68/73 (41.5%/44.5%) left/right, compared with PFX− and controls, in which no hyperintensities were observed (*p* < 0.001). We performed the sensitivity analysis using the pre-consensus subset with concordant ratings of ICP hyperintensities among the two raters and found similar results. Considering all visits, participants with left and/or right ICP hyperintensities at any visit were 0%, 3 (5.6%) and 73 (44.0%) for controls, PFX− and PFX+, respectively. Two of the 3 PFX- participants that showed left and right ICP hyperintensities converted to PFX+ in the following visits. The youngest participant showing ICP hyperintensities was 51.9 years old compared to 48.3 years for the youngest participant with MCP hyperintensities. Left and right ICP hyperintensities displayed high concordance (chi-squared = 316.7, *p* < 0.001 at last visit). Out of the 439 visits from the premutation carriers, ICP hyperintensities were not shown at either side for 311 (70.8%) visits, at both sides for 123 (28.0%), only on the right side for 5 (1.1%) and only on the left side for 0 visits.

### 3.3 Association of ICP hyperintensities with other MRI measures

**FIGURE 2:**
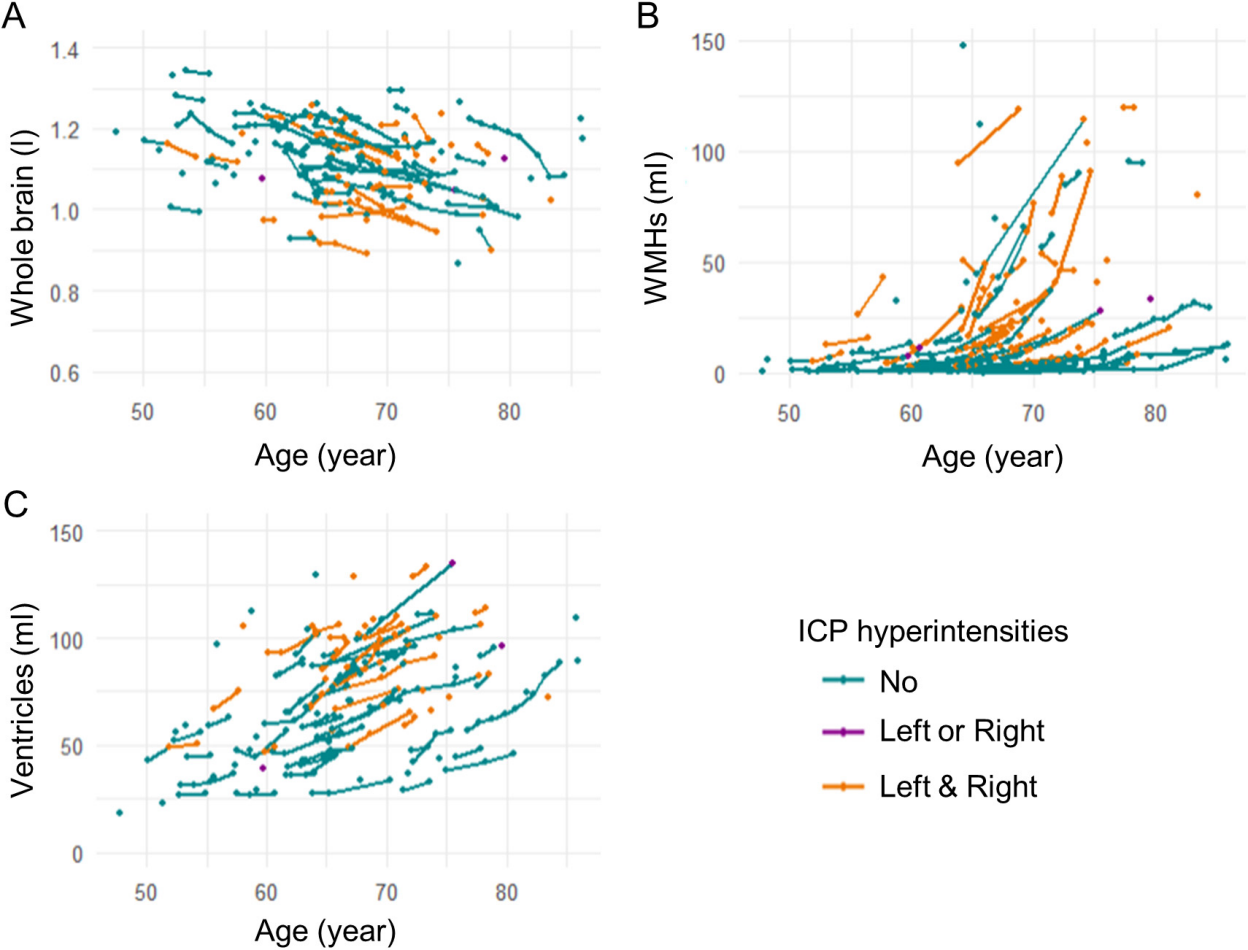
Associations between inferior cerebellar peduncles (ICP) hyperintensities and anatomic volume in PFX+. PFX+ with ICP hyperintensities had **(A)** lower whole brain volume, **(B)** higher WMH volume and **(C)** higher ventricular volume than PFX+ without ICP hyperintensities.

Table 2 shows associations of ICP hyperintensities with whole brain volume, whole brain WMH volume and ventricular volume, adjusted for age, brain scaling factor, scanner software, and head coil, in the PFX+ group. Because of the extremely low occurrences of ICP hyperintensities in the PFX− group, the associations of ICP hyperintensities with other MRI measurements were not performed in PFX−. PFX+ with ICP hyperintensities demonstrated reduced whole brain volume (Figure 2A), increased WMH volume (Figure 2B) and increased ventricular volumes (Figure 2C) compared with PFX+ without ICP hyperintensities at FDR < 0.05. We performed the sensitivity analysis using the pre-consensus subset with concordant ratings of ICP hyperintensities from the two raters. We found that the correlations of the ICP hyperintensities with whole brain volume and ventricular volume were no longer significant while the correlation with WMH volume remained significant (*p* < 0.001). In the sensitivity analysis using the pre-consensus subset acquired after the scanner upgrade and with the 32-head coil, we observed no changes in the results (*p* < 0.043).

**TABLE 2:** Association of inferior cerebellar peduncles (ICP) hyperintensities with other MRI measurements in PFX+.

Correlation	β	SE	CI	*P*	FDR
Whole brain (cm^3^)	*N* = 118, # of observations = 224				
Intercept	0.387	0.068	0.251, 0.522	0	**0**
Age	−0.0053	0.0007	−0.007, −0.004	0	**0**
Left ICP	−0.013	0.006	−0.026, 0	0.043	**0.043**
Intercept	0.384	0.068	0.25, 0.518	0	**0**
Age	−0.0052	0.0007	−0.007, −0.004	0	**0**
Right ICP	−0.015	0.006	−0.027, −0.003	0.018	**0.022**
Whole brain WMH (log mm^3^)	*N* = 185, # of observations = 283				
Intercept	4.686	1.283	2.15, 7.223	0.0004	**0.0006**
Age	0.146	0.007	0.132, 0.16	0	**0**
Left ICP	0.196	0.070	0.057, 0.334	0.006	**0.008**
Intercept	4.905	1.269	2.397, 7.413	0.0002	**0.0003**
Age	0.142	0.007	0.128, 0.156	0	**0**
Right ICP	0.260	0.072	0.118, 0.401	0.0004	**0.0006**
Ventricles (log mm^3^)	*N* = 118, # of observations = 224				
Intercept	8.913	0.299	8.321, 9.505	0	**0**
Age	0.031	0.002	0.027, 0.034	0	**0**
Left ICP	0.040	0.017	0.007, 0.074	0.020	**0.022**
Intercept	8.966	0.299	8.375, 9.558	0	**0**
Age	0.031	0.002	0.027, 0.034	0	**0**
Right ICP	0.034	0.017	0.002, 0.067	0.041	**0.043**

Bold, FDR < 0.05.

In addition, the occurrence of left and right ICP hyperintensities was concordant with left and right MCP hyperintensities (chi-squared = 165.4–177.6, *p* < 0.001). However, more premutation carriers showed hyperintensities in the MCPs than ICPs. While none of the 85 controls showed the MCP sign, 5/54 (9.3%) PFX- and 94/166 (56.6%) PFX+ exhibited left and/or right MCP hyperintensities at any visit. In addition, while no premutation carriers displayed ICP hyperintensities only (ICP without MCP hyperintensities) at any visit, 2/54 (3.7%) PFX- and 35/166 (21.1%) PFX+ showed only MCP hyperintensities at any visit. Among the 2 PFX- carriers who showed only MCP hyperintensities, 1 carrier did not show either MCP or ICP hyperintensities at visit 1 (age 52), exhibited MCP hyperintensities at visits 2–4 (age 58, 60, and 62) and both MCP and ICP hyperintensities at visit 5 (age 65). That carrier’s FXTAS diagnosis converted from “No” at visits 1 and 2 to “Probable” at visit 3 and “Definite” at visits 4 and 5.

### 3.4 The association between ICP hyperintensities and motor and cognitive impairment and psychological symptoms

**FIGURE 3:**
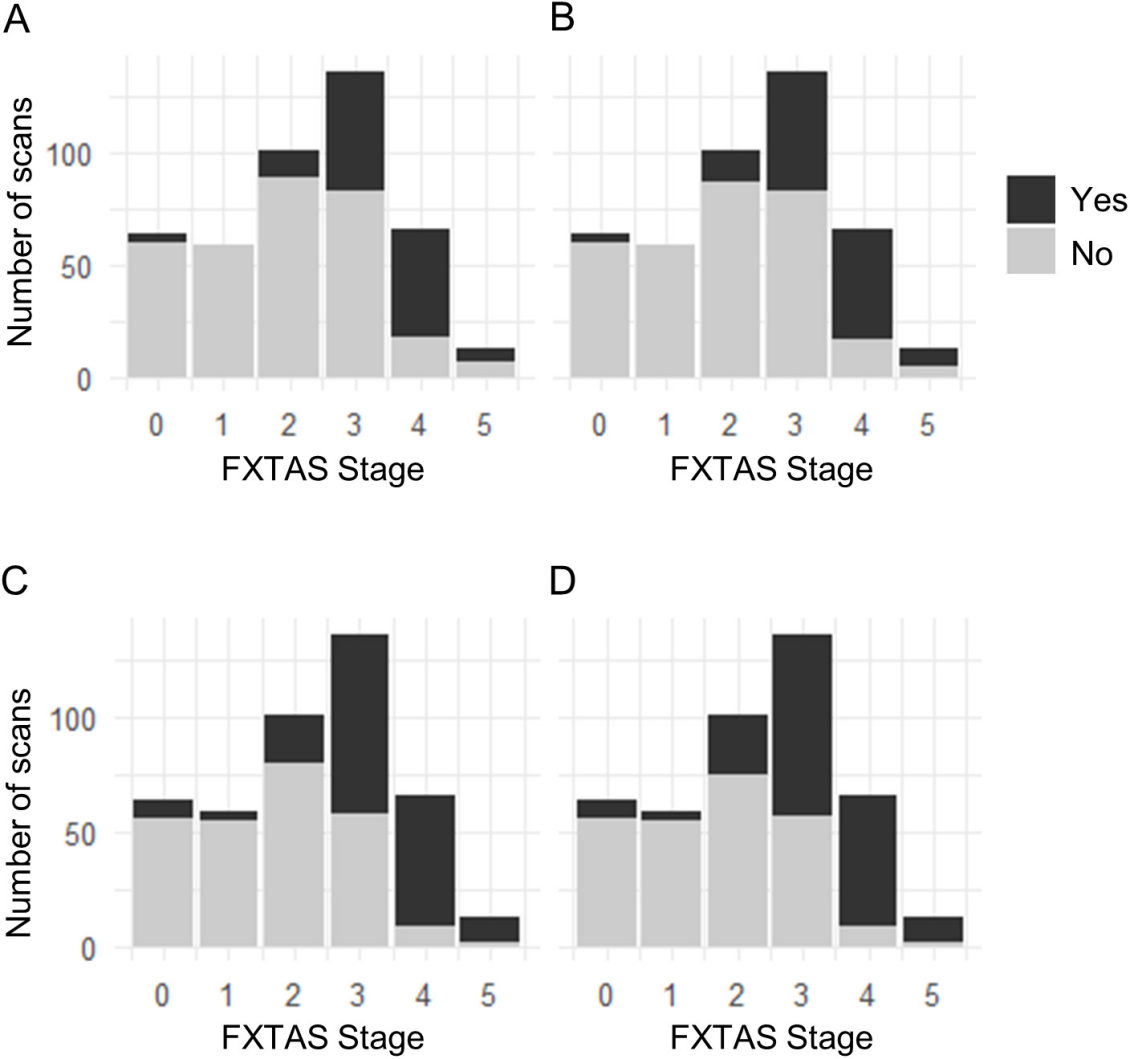
Relationship of the occurrence of inferior cerebellar peduncles (ICP) and middle cerebellar peduncles (MCP) hyperintensities with fragile X-associated tremor/ataxia syndrome (FXTAS) stage. The proportion of scans showing left ICP **(A)**, right ICP **(B)**, left MCP **(C)**, and right MCP **(D)** hyperintensities increases with advances of FXTAS stage.

Table 3 shows the correlation of ICP hyperintensities with motor and cognitive impairment assessed using FXTAS stage, tandem walk, FSIQ, WMI, PSI and BDS-2 scores, and psychological symptoms evaluated using GSI, anxiety and depression scores from SCL-90-R in PFX+. Compared with PFX+ without left or right ICP hyperintensities, PFX+ with left and right ICP hyperintensities experienced more advanced FXTAS stage and PFX+ with right ICP hyperintensities exhibited worse tandem walk impairment and BDS-2 scores adjusted for age, scanner software, and head coil. Figure 3 shows the proportion of scans showing ICP and MCP hyperintensities increases with advances of FXTAS stage. PFX+ with left and right ICP hyperintensities also showed worse FSIQ, WMI, and PSI and faster age-related decline in FSIQ and PSI compared with PFX+ without left or right ICP hyperintensities adjusted for age, years of education, scanner software, and head coil. For psychological symptoms, age-related increase in anxiety scores was faster in PFX+ with left and right ICP hyperintensities while age-related increase in GSI scores approached significance in PFX+ with left ICP hyperintensities compared with PFX+ without left ICP hyperintensities adjusted for age, scanner software, and head coil. In addition, age-related increase in depression scores was faster in PFX+ with left ICP hyperintensities than PFX+ without left ICP hyperintensities.

**TABLE 3:** The correlation of inferior cerebellar peduncles (ICP) hyperintensities with motor and cognitive impairment and psychological symptoms in PFX+.

Comparisons	β	SE	CI	*P*	FDR
FXTAS stage	*N* = 166, # of observations = 342				
Intercept	2.302	0.114	2.077, 2.527	0	**0**
Age	0.071	0.010	0.052, 0.09	0	**0**
Left ICP	0.451	0.092	0.269, 0.633	0	**0**
Intercept	2.271	0.113	2.048, 2.493	0	**0**
Age	0.068	0.009	0.049, 0.086	0	**0**
Right ICP	0.531	0.092	0.349, 0.713	0	**0**
Gait (tandem walk)	*N* = 158, # of observations = 320				
Intercept	1.14	0.10	0.94, 1.34	0	**0**
Age	0.05	0.01	0.035, 0.062	0	**0**
Left ICP	0.13	0.08	−0.037, 0.288	0.13	0.16
Intercept	1.12	0.10	0.92, 1.31	0	**0**
Age	0.05	0.01	0.033, 0.061	0	**0**
Right ICP	0.19	0.08	0.024, 0.355	0.025	**0.039**
Full scale IQ	*N* = 134, # of observations = 227				
Intercept	87.84	6.31	75.4, 100.3	0	**0**
Age	−0.13	0.16	−0.446, 0.189	0.42	0.47
Left ICP	−4.80	2.05	−8.881, −0.722	0.022	**0.036**
Age × left ICP	−0.97	0.28	−1.525, −0.423	0.001	**0.0017**
Intercept	88.18	6.34	75.6, 100.7	0	**0**
Age	−0.12	0.16	−0.442, 0.195	0.44	0.48
Right ICP	−4.78	2.11	−8.98, −0.587	0.026	**0.039**
Age × right ICP	−0.98	0.28	−1.536, −0.417	0.001	**0.0019**
Working memory index	*N* = 131, # of observations = 192				
Intercept	92.62	6.93	78.9, 106.3	0	**0**
Age	−0.21	0.17	−0.555, 0.134	0.23	0.28
Left ICP	−6.67	2.33	−11.34, −1.99	0.006	**0.012**
Intercept	92.92	6.94	79.2, 106.6	0	**0**
Age	−0.20	0.17	−0.542, 0.15	0.26	0.31
Right ICP	−7.05	2.35	−11.7, −2.35	0.004	**0.0083**
Processing speed index	*N* = 105, # of observations = 157				
Intercept	85.07	7.48	70.24, 99.91	0	**0**
Age	0.23	0.21	−0.179, 0.647	0.26	0.31
Left ICP	−7.30	3.07	−13.49, −1.119	0.022	**0.036**
Age × Left ICP	−1.02	0.39	−1.814, −0.233	0.012	**0.022**
Intercept	85.29	7.50	70.42, 100.17	0	**0**
Age	0.23	0.21	−0.184, 0.644	0.27	0.31
Right ICP	−7.25	3.01	−13.32, −1.18	0.020	**0.036**
Age × right ICP	−1.03	0.39	−1.812, −0.244	0.011	**0.021**
BDS-2	*N* = 159, # of observations = 313				
Intercept	18.38	0.69	17.01, 19.75	0	**0**
Age	−0.15	0.05	−0.244, −0.06	0.0014	**0.0031**
Left ICP	−1.12	0.60	−2.298, 0.063	0.063	0.09
Intercept	18.47	0.69	17.01, 19.75	0	**0**
Age	−0.15	0.05	−0.244, −0.06	0.002	**0.0043**
Right ICP	−1.37	0.60	−2.298, 0.063	0.025	**0.039**
SCL-90-R GSI	*N* = 137, # of observations = 266				
Intercept	56.58	1.68	53.26, 59.9	0	**0**
Age	−0.16	0.11	−0.384, 0.056	0.14	0.18
Left ICP	−1.17	1.58	−4.3, 1.963	0.46	0.49
Age × left ICP	0.39	0.19	0.006, 0.769	0.047	0.07
Intercept	56.31	1.67	53.01, 59.61	0	**0**
Age	−0.07	0.10	−0.273, 0.129	0.48	0.51
Right ICP	0.24	1.40	−2.531, 3.003	0.87	0.87
SCL-90-R anxiety	*N* = 137, # of observations = 266				
Intercept	55.17	1.68	51.84, 58.50	0	**0**
Age	−0.22	0.11	−0.431, 0.001	0.051	0.073
Left ICP	−3.02	1.58	−6.152, 0.121	0.06	0.08
Age × left ICP	0.65	0.19	0.267, 1.035	0.001	**0.0023**
Intercept	55.12	1.69	51.78, 58.45	0	**0**
Age	−0.20	0.11	−0.422, 0.016	0.069	**0.093**
Right ICP	−2.75	1.62	−5.95, 0.46	0.093	0.12
Age × right ICP	0.56	0.20	0.177, 0.951	0.005	**0.0092**
SCL-90-R depression	*N* = 137, # of observations = 266				
Intercept	57.96	1.88	54.24, 61.69	0	**0**
Age	−0.21	0.13	−0.478, 0.051	0.11	0.14
Left ICP	−0.84	0.98	−2.772, 1.097	0.39	0.44
Age × left ICP	0.26	0.11	0.034, 0.488	0.025	**0.039**
Intercept	57.34	1.87	53.64, 61.05	0	**0**
Age	−0.03	0.11	−0.245, 0.18	0.76	0.78
Right ICP	0.26	0.87	−1.454, 1.971	0.77	0.78

Bold, FDR < 0.05.

We performed the sensitivity analysis using the pre-consensus subset of concordant ICP hyperintensity ratings from the two raters. The results remained the same for FXTAS stage, FSIQ, WMI, GSI, and depression (*p* < 0.05) while the negative effect of left ICP hyperintensities on gait, BDS, and anxiety became significant (*p* < 0.05) and the effect of left and right ICP on PSI became non-significant (*p* > 0.05). The sensitivity analysis using the subset acquired after the scanner upgrade and with the 32-channel head coil showed no changes in the results for FXTAS stage, FSIQ, WMI, PSI, GSI, anxiety and depression except for the interaction between right ICP and gait that became non-significant (*p* > 0.05).

### 3.5 Correlation of ICP hyperintensities with FMR1 molecular measures

Among the 202 premutation carriers, seven carriers (3%) showed CGG size mosaicisms (i.e., unmethylated allele varying in size within the premutation range). Of these seven carriers, one showed a CGG smear ranging from approximately 110–130 repeats, and six carriers had two discrete premutation alleles with the smaller alleles ranging from 52 to 83 CGG repeats and the larger alleles ranging from 66 to 105 CGG repeats. The difference between the two alleles ranged from 12 to 28 CGG repeats. The premutation carrier showing a CGG smear was diagnosed of FXTAS at his second visit while the remaining six carriers with two alleles were diagnosed of FXTAS at all visits. Table 4 shows the correlation of ICP hyperintensities with CGG repeat length at last visit and *FMR1* mRNA including all visits in PFX+. PFX+ with left and right ICP hyperintensities at last visit showed larger CGG repeats than PFX+ without left or right ICP hyperintensities adjusted for age, scanner software, and head coil. Using the largest allele for the seven carriers with CGG size mosaicism rather than the averaged CGG repeat length, the correlation of left and right ICP hyperintensity ratings with CGG repeat size remained significant (*p* = 0.005). We perform the sensitivity analysis using the subset with concordant ICP hyperintensity ratings and the subset acquired after the scanner upgrade and with a 32-channel head coil (*p* ≤ 0.004). We found no changes in our results (*p* ≤ 0.004). The shortest CGG repeat lengths showing ICP and MCP hyperintensities were 78 and 77 repeats, respectively. In contrast, mRNA levels were not significantly difference between PFX+ with and without ICP adjusted for age, scanner upgrade, and head coil.

**TABLE 4:** The correlation of inferior cerebellar peduncles (ICP) hyperintensities with Fragile X Messenger Ribonucleoprotein 1 (*FMR1*) molecular measurements in PFX+.

Correlation	β	SE	CI	*P*
**CGG repeat length (last visit) (*N* = 163)**				
Intercept	97.17	3.58	90.09, 104.25	<0.001
Age	−0.54	0.17	−0.87, −0.21	0.0013
Left ICP	7.09	2.48	2.19, 11.98	0.0048
Intercept	97.17	3.58	89.6, 104.0	<0.001
Age	−0.54	0.17	−0.87, −0.22	0.0013
Right ICP	7.09	2.48	2.21, 11.9	0.0048
**FMR1 mRNA (all visits) (*N* = 153, # of observations = 288)**				
Intercept	1.007	0.032	0.94, 1.07	0
Age	−0.004	0.002	−0.0078, 0.0007	0.10
Left ICP	0.047	0.029	−0.012, 0.105	0.12
Intercept	1.005	0.032	0.94, 1.07	0
Age	−0.004	0.002	−0.008, 0.0005	0.09
Right ICP	0.052	0.029	−0.0059, 0.11	0.08

### 3.6 Time-varying relationship with FXTAS stage

**FIGURE 4:**
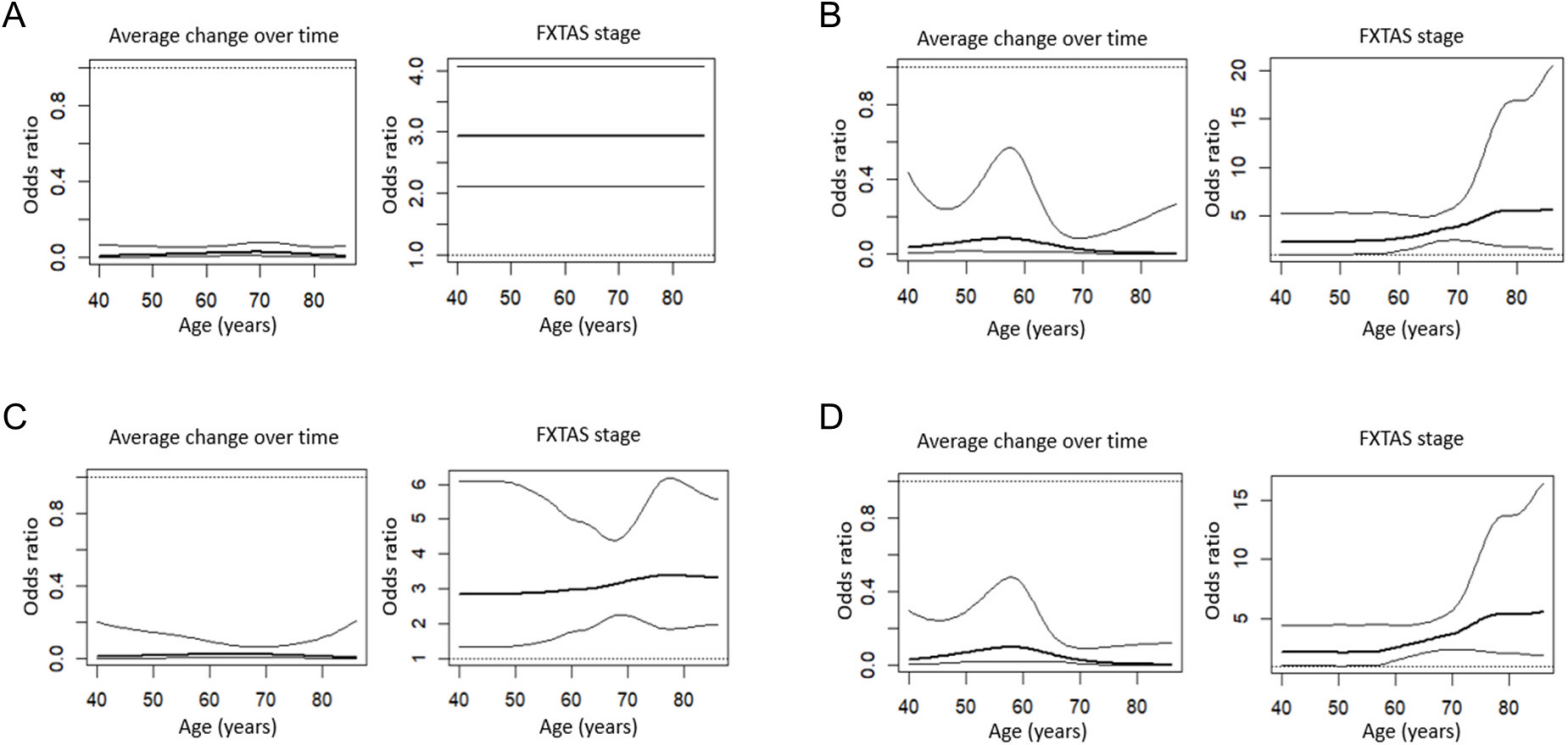
Relationships between inferior cerebellar peduncles (ICP)/middle cerebellar peduncles (MCP) hyperintensities and fragile X-associated tremor/ataxia syndrome (FXTAS) stage over the age range. Relationship between left **(A)** and right **(C)** ICP hyperintensities and FXTAS stage over age. Relationship between left **(B)** and right **(D)** MCP hyperintensities and FXTAS stage over age.

We further explored changes in the relationships of ICP/MCP hyperintensity with FXTAS stage over the age range (40–85 years) in the 202 premutation carriers using time-varying effect models (Figure 4). The relationship between ICP and FXTAS stage remained constant over the age range while the relationship between MCP and FXTAS stage became stronger as age advanced.

## 4 Discussion

In order to demonstrate if the ICPs were involved in FXTAS pathophysiology, we visually assessed the presence or absence of ICP hyperintensities in three groups: PFX+, PFX- and non-premutation carriers (controls). The results demonstrated that 41.5% and 44.5% PFX+ showed left and right ICP hyperintensities, respectively, while none of the controls or PFX− exhibited ICP hyperintensities at last visit. Compared with PFX+ without ICP hyperintensities, PFX+ with ICP hyperintensities demonstrated (1) more severe brain atrophy and white matter disease, more enlarged ventricles, and increased likelihood to show the MCP sign; (2) higher FXTAS stage, more impaired gait and executive dysfunction, slower processing speed, and faster age-related decline in processing speed; (3) faster age-related increase in anxiety and depression symptoms, and (4) longer CGG repeat length. Previous studies have mainly focused on the MCP involvement in FXTAS. This study adds evidence that the tracts within ICPs are also affected in FXTAS and that its occurrence is associated with MRI measures of neurodegeneration, impairment in motor and cognition, psychological symptoms and *FMR1* molecular measurements. However, because ICP hyperintensities were always accompanied by MCP hyperintensities, these associations cannot be attributed to ICP pathology independently, and may reflect a more advanced or global disease state.

The fact that ICP hyperintensities were observed in 44.0% of the PFX+ group, compared to only 5.6% of PFX- individuals, and that no controls showed hyperintensities at any visit, suggests a strong association between ICP hyperintensities and FXTAS pathophysiology. Previous studies have largely focused on white matter degeneration, especially in the MCPs of premutation carriers with or without FXTAS (Filley et al., 2015), suggesting that white matter pathology is the primary initiating factor, and proposing FXTAS as a primarily white matter disease. Our results align with these previous findings and add further evidence that the white matter within the ICPs is also affected in FXTAS.

The term “ICP sign” was recently introduced as a specific imaging marker for differentiating multiple system atrophy with predominant cerebellar ataxia (MSA-C) from spinocerebellar ataxias, particularly in early stages (Lim et al., 2024). ICP hyperintensities showed strong concordance with MCP hyperintensities. Only 3.7% of PFX- and 21.1% of PFX+ showed isolated MCP hyperintensities, while no premutation carriers exhibited isolated ICP hyperintensities. Prior evidence suggests that MCP signal abnormalities may appear in asymptomatic *FMR1* premutation carriers before FXTAS onset, indicating that MCP involvement may precede symptoms (Filley et al., 2015; Famula et al., 2018). In our study, ICP hyperintensities always accompanied MCP hyperintensities, but MCP hyperintensities sometimes occurred alone. This suggests a possible temporal sequence, with MCP abnormalities potentially appearing first. However, visually assessing the ICP hyperintensities is challenging due to the tract’s narrow width and close anatomical proximity to the MCP. Therefore, the qualitative visual assessment of ICP hyperintensities may underestimate early pathology and could bias interpretation of the temporal relationship between MCP and ICP involvement. Furthermore, temporal inferences regarding MCP and ICP involvement should be considered hypothesis-generating, as the observational nature of the study precludes causal conclusions about disease progression. Future longitudinal studies using quantitative and advanced MRI approaches, for example, tract-specific diffusion analyses or quantitative FLAIR signal measurements, could overcome the qualitative visual assessment limitations and provide more objective assessments of cerebellar white-matter integrity and aid in interpreting the temporal relationship between MCP and ICP involvement.

Importantly, ICP hyperintensities correlated with reduced whole brain volume, increased WMH volume, and increased ventricular volume in PFX+, consistent with prior findings of reduced brain volume and accelerated ventricular enlargement in PFX− (Wang et al., 2017). Evidence supports that brain volume loss in premutation carriers occurs before FXTAS develops, suggesting other mechanisms beyond WMHs appearance (Cohen et al., 2006). Ventricular enlargement, reflecting dysfunctional CSF dynamics (Yamada et al., 2021) and brain atrophy (Apostolova et al., 2012), is common in aging and neurodegenerative diseases like Alzheimer’s, Parkinson’s, and vascular dementia (Missori and Currà, 2015). In our study, white matter damage in the cerebellar peduncles, which are adjacent to the fourth ventricle, may contribute to the observed ventricular enlargement. Although enlargement of the fourth ventricle has been reported in PFX−, ICP hyperintensities are not typically present. Still, this does not exclude the possibility of underlying ICP damage in PFX−, as such damage may not manifest as visible hyperintensities. Advanced MRI techniques, such as diffusion MRI, may offer greater sensitivity to structural alterations not detectable with T2-FLAIR.

Furthermore, we found significant associations between ICP hyperintensity ratings and motor dysfunction such as FXTAS stage and tandem walk in PFX+. These findings suggest that the ICPs may contribute to the ataxia observed in FXTAS, as they contain fiber tracts from the vestibular and visual systems, as well as proprioceptive pathways (Snell, 2010; Jones et al., 2013; Paracuellos-Ayala et al., 2025). These results align with a previous study showing that the MCP sign in premutation carriers (with and without FXTAS) was associated with motor and cognitive impairment (Wang et al., 2022). However, we could not determine whether the associations between ICP hyperintensities, motor dysfunction, and FXTAS severity were independent of the MCP sign because of the high correlation between the occurrence of MCP and ICP hyperintensities.

Further findings are the associations between ICP hyperintensities and lower cognitive function, as measured by BDS-2, WMI, and PSI, and faster age-related decline in IQ and processing speed in PFX+. Although the relationship between cognitive dysfunction and CP’s alterations remains poorly understood, it appears to stem from disrupted cerebellar access to cognitive and limbic afferent information. Prior studies have identified associations between cognitive impairment and abnormalities in the SCP and MCP in multiple sclerosis (Nicoletti et al., 2017; Tobyne et al., 2017), and schizophrenia (Okugawa et al., 2006). In FXTAS, cognitive impairment is thought to result, at least partially, from white matter disruption affecting fibers that carry prefrontal information through the anterior corpus callosum and the MCP (Stoodley and Schmahmann, 2010; Filley et al., 2015). The ICPs transmit proprioceptive, vestibular, and integrated somatosensory information from the reticular system and inferior olive, potentially contributing to motor learning and error correction. However, no studies have demonstrated a direct link between isolated ICP damage and cognitive dysfunction. While ICP hyperintensities might contribute to cognitive impairment in FXTAS, our study found an association between MCP and ICP hyperintensities, suggesting ICP alterations alone may not fully account for cognitive dysfunction.

Interestingly, our study revealed that PFX+ with ICP hyperintensities experienced a more rapid age-related increase in anxiety and depression scores, compared to PFX+ without ICP hyperintensities. Emotional processing (especially fear response) involves multiple brain regions, with evidence that the cerebellum, especially the posterior lobes and vermis, plays a contributing role (Ciapponi et al., 2023). Noradrenergic and serotonergic fibers from the locus coeruleus and raphe nuclei, which contribute to fear-related emotions, are thought to project to the cerebellum via the MCPs (Jones et al., 2013). However, these pathways, along with other aspects of emotional processing, remain incompletely characterized in humans, and the role the ICP might play in emotional responses is unclear.

Our finding suggests that the ICPs may influence cerebellar emotional processing. However, the observed age-related increase in anxiety and depression scores is unlikely to solely result from ICP involvement. Comorbidities and the psychological impact of neurodegeneration, which are prevalent in various neurodegenerative disorders (Boeschoten et al., 2017), including FXTAS, also contribute significantly to heightened distress. Moreover, premutation carriers often exhibit elevated depression and anxiety scores even before the onset of motor symptoms in FXTAS (Tassone et al., 2023). Given that ICP hyperintensities in our study were associated with advanced FXTAS stages, it remains uncertain whether these results arise directly from ICP and MCP damage or reflect the broader disease burden in FXTAS.

In PFX+, we observed that those with ICP hyperintensities at their last visit had larger CGG repeat expansions compared to those without ICP hyperintensities. At the molecular level, FXTAS is caused by a toxic gain-of-function mechanism in which the expansion of CGG repeats in the *FMR1* gene in the premutation range results in excessive *FMR1* mRNA (Mila et al., 2018). This excessive mRNA disrupts normal cellular functions leading to neuronal dysfunction and cellular toxicity. Our findings are consistent with prior studies linking larger CGG repeat expansions to MRI abnormalities, including regional and whole-brain atrophy, ventricular enlargement, and increased WMH volume (Loesch et al., 2005; Cohen et al., 2006; Wang et al., 2017). Similarly, reduced fractional anisotropy in the MCP (Hashimoto et al., 2011), and decreased connectivity strength in the SCP (Wang et al., 2013), have been associated with CGG repeat expansions in the premutation range. Higher *FMR1* mRNA levels have also been negatively correlated with SCP connectivity strength (Wang et al., 2013). Interestingly, one study found that higher *FMR1* gene methylation levels correlated with decreased cerebellar peduncle microstructure (lower mean diffusivity) in the MCP and ICP of female premutation carriers without FXTAS (Shelton et al., 2017). Our results strengthen previous evidence linking molecular alterations to cerebellar peduncle microstructure in premutation carriers with and without FXTAS (Shelton et al., 2017), and further demonstrate an association between *FMR1* molecular measurements and ICP T2-FLAIR hyperintensities in FXTAS.

The main limitation of this study is the qualitative assessment of ICP hyperintensities through visual MRI analysis, which lacks the precision of quantitative methods. Although inter-rater reliability was good, it was lower for the ICPs compared with the MCPs, reflecting the technical challenges of visually assessing this tract. Furthermore, because the MCPs and ICPs were assessed on the same T2-FLAIR images, readers could not be blinded to MCP signal abnormalities when visually rating ICP hyperintensities, which may represent an inherent limitation of this visual assessment approach. Finally, restricting the samples to male premutation carriers aged 40 years or older limits the generalizability of our findings, which may not extend to female carriers or to earlier, preclinical stages of premutation-associated brain involvement. However, in our cohort of 202 male premutation carriers, the youngest carrier showing ICP/MCP hyperintensities was 51.9/48.3 years old, indicating the limited utility of using ICP/MCP hyperintensities for FXTAS diagnosis or prognosis in male premutation carriers younger than 40 years.

To sum up, the current study investigates ICP T2-hyperintensity in male premutation carriers and identifies increased ICPs hyperintensities in PFX+ compared to controls and PFX-. Furthermore, 2 out of 3 PFX− with ICPs hyperintensities converted to PFX+ in the following visits and ICPs hyperintensities in PFX+ were associated with other MRI features of neurodegeneration, FXTAS stage, poorer motor and cognitive function, higher age-related psychological distress and increased CGG repeat length compared with PFX+ without ICPs hyperintensities. In conclusion, this study provides valuable insights into: (1) the understanding of WMHs in FXTAS, revealing a new location of abnormal T2-weighted MRI signal in the ICP, (2) motor dysfunction in FXTAS, adding some evidence that fiber tracts from the vestibular and visual systems, and proprioceptive pathways might also be involved in FXTAS, and (3) reinforcing the association of CGG expansion in the range of brain changes seen in FXTAS.

## Data Availability

The raw data supporting the conclusions of this article will be made available by the authors, without undue reservation.
